# Biomechanical Properties of Knee Medial Collateral Ligament Compared to Palmaris Longus for Ulnar Collateral Ligament Reconstruction

**DOI:** 10.1007/s10439-023-03188-z

**Published:** 2023-04-19

**Authors:** Dave Huang, Lukas Foster, Michael Stone, David Kulber, Melodie F. Metzger

**Affiliations:** 1 Orthopedic Biomechanics Laboratory, Cedars-Sinai Medical Center, Los Angeles, CA USA; 2 Department of Orthopedics, Cedars-Sinai Medical Center, Los Angeles, CA USA

**Keywords:** UCL reconstruction, Palmaris longus, Knee medial collateral ligament, Graft

## Abstract

Ulnar collateral ligament reconstruction (UCLR) is frequently performed among injured overhead-throwing athletes. One of the most common graft choices when performing a UCLR is the ipsilateral palmaris longus tendon (PL). The purpose of this study was to investigate the material properties of aseptically processed cadaveric knee collateral ligaments (kMCL) as a potential graft source for UCLR and compare them to the gold standard PL autograft. Each PL and kMCL cadaveric sample was subjected to cyclic preconditioning, stress relaxation, and load-to-failure testing, and the mechanical properties were recorded. PL samples exhibited a greater average decrease in stress compared to the kMCL samples during the stress-relaxation test (*p* < 0.0001). PL samples also demonstrated a greater average Young’s modulus in the linear region of the stress–strain curve compared to the kMCL samples (*p* < 0.01). The average yield strain and maximum strain of kMCL samples were significantly greater than the PL, *p* = 0.03 and 0.02, respectively. Both graft materials had comparable maximum toughness and demonstrated a similar ability to deform plastically without rupture. The clinical significance of our result is that prepared knee medial collateral ligament allografts may provide a viable graft material for use in the reconstruction of elbow ligaments.

## Introduction

The ulnar collateral ligament (UCL) is the primary soft tissue stabilizer against valgus stress in the elbow, making it prone to injury during overhead throwing motions. Tensile failure of the UCL leads to valgus instability, pain, loss of overhead throwing velocity, and accelerated joint degeneration. Dr. Frank Jobe first described the successful reconstruction of the UCL in 1986, allowing athletes to return to their pre-injury level of the sport [19,20]. Since then, a variety of ulnar collateral ligament reconstruction (UCLR) techniques have been described, including methods that use both allograft and autograft tendons [12,22,23].

The palmaris longus (PL) autograft has become the gold standard for graft selection in the UCLR of the elbow, although there is no data to suggest it is mechanically superior compared to other graft options [3,7]. The popularity of PL autografts likely stems from the historical assumption that the PL muscle–tendon unit contributes minimally to our upper extremity function [18]. However, a recent study on baseball players contradicts this assumption by demonstrating that the dominant PL is indeed active during throwing motions and is significantly larger than the non-dominant PL. Thus, the PL may be more important than originally thought and might not make an optimal graft material, particularly in professional athletes [25]. Alternative UCLR autografts include knee hamstring tendons with reported similar return to play rates as PL autografts of approximately 80%, and variable failure rates ranging from 0 to 7.1% [14,32].

Regardless of the autograft used, graft harvest adds complexity and increased risk to the surgery. Superficial infection, pain, and dysfunction at the graft site, hamstring muscle weakness, and accidental median nerve damage during PL tendon harvest have all been described [21,30]. To minimize these risk, allografts, connective tissue harvested from cadaveric donors, have been used with similar functional outcomes [21]. While concerns regarding sterilization, added procedural costs, delayed graft incorporation, and disease transmission are associated with allograft tissue, improved preparation and surgical techniques are increasing interest in using allograft materials for ligament reconstruction procedures.

Tendons are typically harvested for grafts when performing ligament reconstructive surgeries because they can be repurposed without significant loss of function due to redundancy within muscle compartments. Tendons are also long enough to recreate a ligament, thread through bone tunnels, and anchor to bone for reliable fixation [2]. Historically, ligament allografts have not been used as they typically do not provide adequate length. As a result, tendons and ligaments have become interchangeable and are often treated as identical tissues despite having known differences in viscoelastic behavior [14].

The collateral ligaments of the knee have recently been described as an alternative graft source for small joint reconstruction in the hand [31]. Knee collateral ligaments may also provide an alternative graft choice for reconstruction of the elbow UCL as they meet the length requirement for standard UCLR techniques [5]. Theoretically, by replacing “like material with like,” ligament allografts can potentially provide improved biomechanical properties that better recreate the biologic structure of native ligaments. The purpose of this study was to measure and compare the biomechanical properties of fresh frozen knee medial collateral ligament (kMCL) allografts and compare them to the gold standard PL tendon autografts as a potential graft choice for UCL reconstruction.

## Materials and Methods

### Tissue Preparation

PL tendon grafts were harvested from eight male fresh frozen cadaveric mid-humerus to finger specimens (all male 48 ± 11.5 years; range 30–64 years) acquired from an institute-approved tissue bank (Science Care, CA). The palmaris tendon was excised between the PL muscle–tendon interface and palmar aponeurosis. From this sample, a 5 × 50 mm measured section was dissected from the mid-substance of the tendon. Previous research has demonstrated there is no significant difference in the material properties of the distal, proximal, and middle sections of the PL tendon [28].

Aseptically processed kMCL allografts (all male 25.9 ± 3.4 years; range 21–31) were also acquired from a tissue bank (MTF Biologics, Edison, NJ). In the same manner as harvesting the PL tendons, 5 mm × 50 mm sections were carefully dissected from the central region of the MCL allografts following the longitudinal direction of the fibers using a scalpel. Both PL tendon and kMCL samples were covered with phosphate‐buffered saline (PBS) soaked gauze individually and stored at − 30 °C prior to mechanical testing.

Grafts were thawed at room temperature while wrapped in PBS soaked gauze. A 20 mm gauge length of each kMCL and PL test sample was marked with a skin marker and the remaining 15 mm ends were secured between sandpaper using cyanoacrylate. The thickness and width of each sample was measured with a digital caliper to calculate the elliptical cross-sectional area. While the cyanoacrylate was setting, the exposed sample was covered with PBS soaked gauze to prevent drying.

### Mechanical Testing

**Fig. 1:**
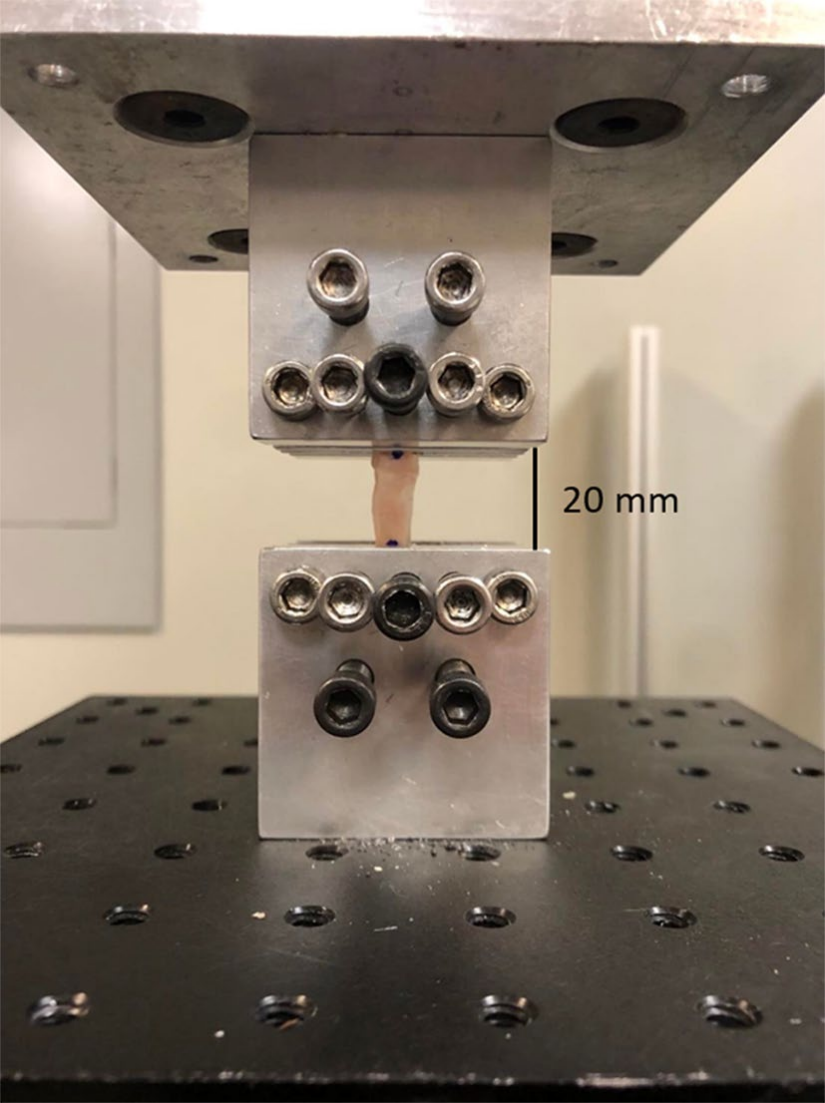
Mechanical testing set-up

Once prepared, each sample was mounted onto a customized tensile testing jig with the 20 mm gauge section exposed, Fig. 1. Each sample was clamped between serrated metal plates via bolts that were torqued to 1 Nm using a manual torque wrench. The jig was secured to the frame of the hydraulic mechanical testing system (370.02 Bionix, MTS Systems Corp., Eden Prairie, MN). A 1 N tensile preload was applied to the sample, followed by 10 cycles of preconditioning between 1.5 and 4.5% engineering strain at 0.5 Hz. Afterward a stress-relaxation test was performed by applying a 5% engineering strain for 300 s while the change in force was recorded. This was followed by load to failure at a rate of 1% strain per second [27]. The test automatically stopped when the sample ruptured, indicated by a 30% drop in load. Displacement and tensile force were continually recorded.

**Fig. 2:**
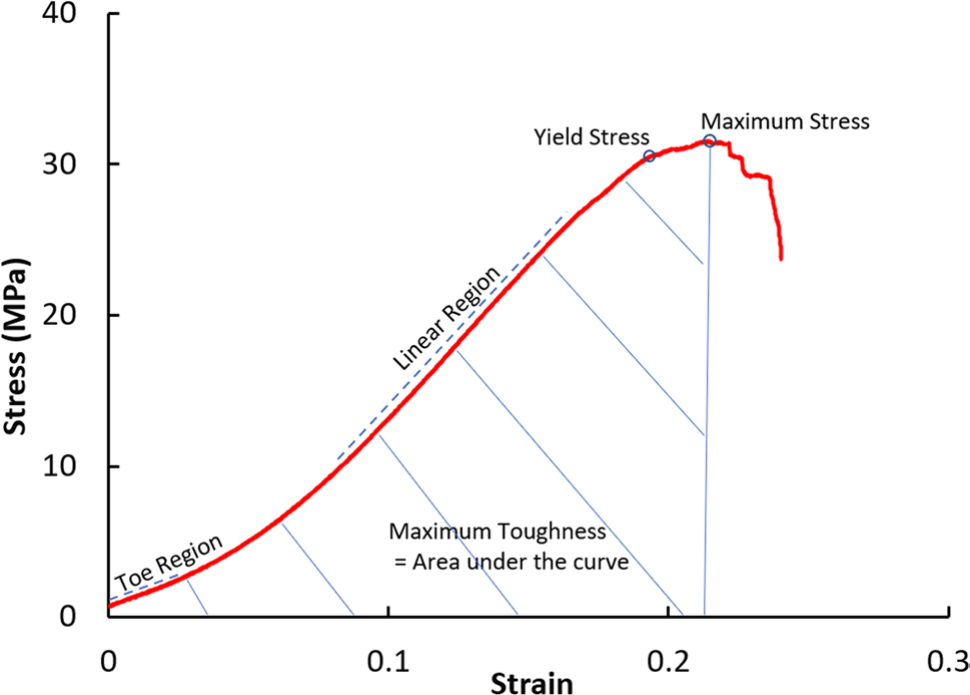
Stress–strain graph of one of the kMCL samples depicting outcomes measures analyzed, including: toe region Young’s modulus (slope of the toe region line), linear region Young’s modulus (slope of the linear region line), yield stress, maximum stress, and maximum toughness (the area under the curve)

Outcome measures were calculated using a customized script (Python, Python Software Foundation, Wilmington, DE): yield stress, yield strain, maximum stress, maximum strain, yield toughness, maximum toughness, toe region Young’s modulus, linear region Young’s modulus, and percent stress-relaxation. Stress $\left(\sigma = \frac{\mathrm{Tensile \,Force}}{\mathrm{Cross\, Sectional\, Area}}\right)$ and strain $\left(\varepsilon = \frac{\mathrm{Displacement}}{\mathrm{Gauge \,Length}}\right)$ were converted from tensile force and displacement. Toe region and linear region Young’s moduli $\left(E=\frac{\mathrm{Change\, in\, Stress}}{\mathrm{Change\, in\, Strain}}\right)$ were defined visually from the slopes of the stress–strain curve with a corresponding *R*^2^ coefficient of 0.99 to validate the goodness of the fit. Toughness was obtained by calculating the area under the stress–strain curve between the maximum and minimum stress values. Yield point was defined by the points at which the *R*^2^ value decreased from 0.99, corresponding to where the elastic behavior ended and plastic behavior began, Fig. 2. Percent stress-relaxation was calculated from the change in stress during the 300-second loading protocol, percent stress-relaxation $=\frac{{\sigma }_{{t}=0 }- {\sigma }_{t=300}}{{\sigma }_{{t}=0}}*100\%.$

### Statistical Analysis

Normalcy was confirmed using the Kolmogorov–Smirnov test. kMCL and PL grafts were compared using the Student *t*-test. Data is presented as means and standard deviations with significance set at *p* < 0.05.

## Results

All 16 specimens (8 PL, 8 kMCL) ruptured at the mid-substance during tensile testing and the results from all specimens were included in the analysis. The mean age of cadavers used to create PL graft samples was significantly greater than the age of cadavers used to create knee MCL allografts, *p* < 0.0001.

**Fig. 3:**
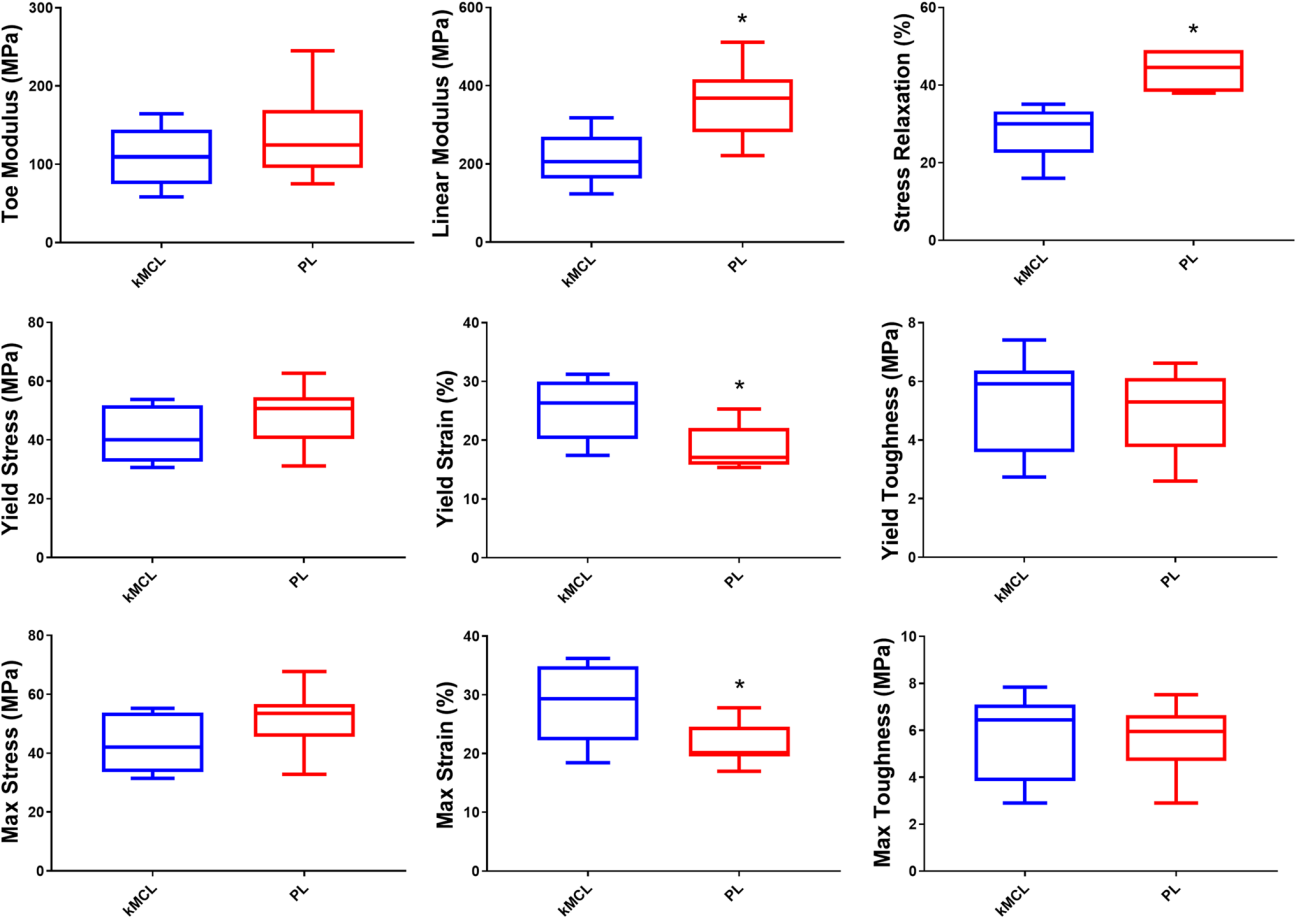
Material properties measured during stress-relaxation and failure test of the PL and knee medial collateral ligament (kMCL)

**Fig. 4:**
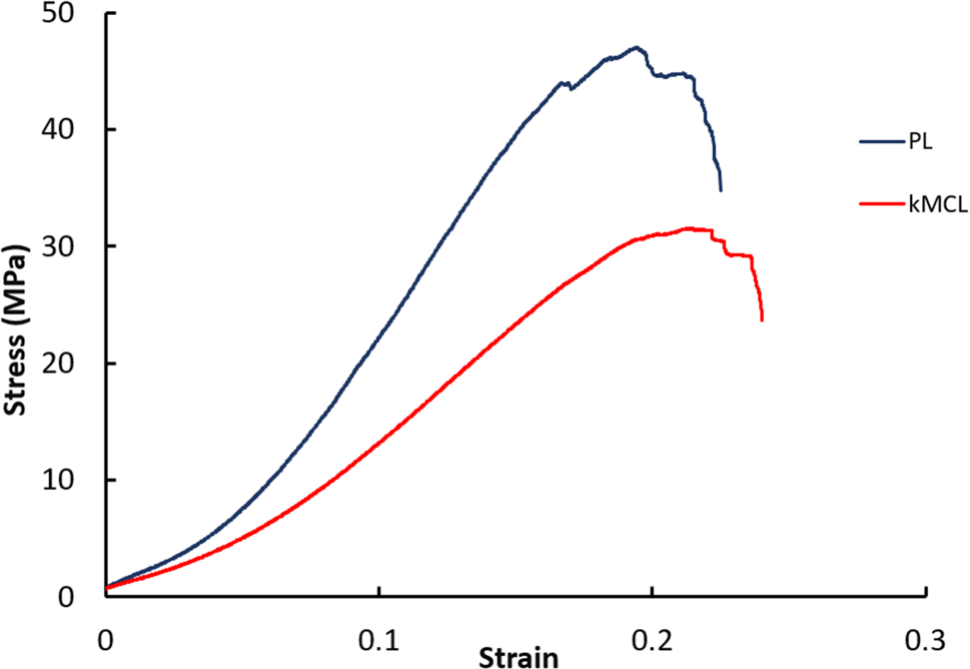
Representative stress–strain graph of the two different graft materials PL (palmaris longus) and knee medical collateral ligament (kMCL), demonstrating typical toe, linear, and failure region of soft biological tissues

PL samples had a greater average percent decrease in stress (43.9%) compared to kMCL samples (28.0%) during the stress-relaxation test, *p* < 0.0001, Fig. 3. The PL and kMCL samples both exhibited typical soft tissue stress–strain curves during load to failure testing, Fig. 4. There was no difference in the modulus of the toe region between the two graft materials, *p* = 0.29. The average modulus of the linear region of PL samples was significantly greater than kMCL samples, *p* < 0.01. The average yield strain and maximum strain of kMCL samples was significantly greater than that measured for PL samples, *p* = 0.03 and 0.02, respectively. There were no significant differences in yield stress, maximum stress, yield toughness, and maximum toughness between the two graft materials.

## Discussion

This study quantified and compared the mechanical properties of kMCLs to PL tendons as a potential new graft material for reconstruction of the UCL. In general, the mechanical behavior of tendons and ligaments are qualitatively similar, with subtle, yet important differences based on collagen orientation, composition, and functionality [5]. Our results are consistent with these previously described differences. PL autografts were significantly stiffer than kMCL allografts, likely due to greater alignment of tendon versus ligament tissue [5]. In addition, both maximum and yield strains were significantly greater in kMCL compared to PL specimens, which may be due to the greater elastin content of ligaments compared to tendons [5,13,15].

In the present study, PL tendon samples demonstrated greater percent stress-relaxation compared to kMCL samples. Stress-relaxation is highly dependent on the amount of strain applied with distinct differences between tendons and ligaments. For instance the rate of stress relaxation decreases with increasing strain for ligaments, but has shown to increase with increasing strain for tendons [6,9–11,16]. The strain used in the present study was 5%, which demonstrated a similar decrease in stress compared to previous findings for both PL and kMCL [6,28].

Knee MCL samples elongated an average 25% of their original length before plastic deformation which was significantly greater than PL samples at an average 19%. Additionally, both PL and kMCL samples demonstrated a similar ability to deform plastically without rupture, both withstanding approximately 3% additional elongation prior to rupture. While there was a significant difference in the linear Young's modulus between the two groups, both samples had comparable maximum toughness, defined as a material’s resistance to rupture [24].

The hamstring gracilis (GR) tendon is another common autograft harvested for UCLR. Previous studies have evaluated the biomechanical properties of the native UCL bundles, PL, and GR, Table 1. Solon et al., tested PL and GR samples and demonstrated similar PL tendon stress relaxation to the data presented in our study, but their linear and toe region modulus were both lower. This is likely because our calculation of the toe region modulus was after the stress-relaxation precondition, resulting in tendon and ligament fibers that were not fully relaxed. Our data was therefore captured later in the non-linear toe region where the tangent begins to increase toward that of the linear portion of the stress–strain curve [1]. Furthermore, Solon et al. sectioned the thickness of the samples to 0.6 mm using a metal thickness gauge. In our study, the thickness of the samples was preserved to their native full thickness, and it is possible that sectioning induced some of the difference in moduli between studies. Regardless, the toe region is likely not an important mechanical factor as it is unclear whether this slack region exists in vivo [5].

**Table 1:** Comparison of the native UCL bundles, PL, GR, and kMCL graft

Specimen	Author (years)	*N*	Age	Percent stress relaxation	Toe region Young’s modulus (MPa)	Linear region Young's modulus (MPa)
PL	This study	8 (8 M)	44 ± 5	43.9 ± 5.0	136.6 ± 54.8	358.7 ± 92.3
kMCL	This study	8 (8 M)	28 ± 7	28.0 ± 6.5	110.4 ± 38.2	213.8 ± 63.7
PL	Solon et al. [28]	13 (8 M; 5 F)	65 ± 9	36.6 ± 9.8	10.5 ± 8.8	192.2 ± 120.6
GR	Solon et al. [28]	11 (4 M; 7 F)	44 ± 6	38.4 ± 10.1	5.5 ± 5.4	117.8 ± 80.9
Native UCL—anterior bundle	Smith et al. [27]	34 (16 M; 18 F)	67 ± 17	34.5 ± 8.7	3.6 ± 3.0	23.2 ± 23.2
Native UCL—posterior bundle	Smith et al. [27]	34 (16 M; 18 F)	67 ± 17	37.7 ± 15.1	1.5 ± 2.9	5.6 ± 10.4

Differences in moduli may also be due to the increased age of PL specimens used in Solon et al. compared to the present study. Previous studies demonstrate that the strength of ligaments and tendons generally decreases with age, but there is conflicting data on whether age effects the elastic modulus and these changes are likely tissue specific [4,5,26,29]. The sex distribution of samples was also different between the Solon study (8 M; 5 F) and the present study (8 M), although previous studies suggested that sex-based difference in graft material are minimal [4,8].

Knee MCL samples had lower stress relaxation and greater Young’s modulus compared to the native UCL, it may be due to that Smith et al. tested the entire anterior and posterior bundles by exercising the tissue from their origins, which may not be comparable with the samples that were dissected from the mid-substance [27].

Our results demonstrate several favorable biomechanical properties of prepared kMCL allografts as a potential graft source for UCLR. The reduced change in stress relaxation exhibited by kMCL compared to PL tendons suggests a slower reduction in graft tension when held at a constant strain. This factor may be important when pre-tensioning the graft at the time of surgery as inadequate tensioning can lead to laxity of the final construct. PL grafts were approximately twice as stiff as kMCL. It is unclear how this difference in stiffness may affect clinical performance of kMCL, although reconstruction with ligament over tendon may result in improved kinematics. Additionally, PL samples had significantly lower yield strain and maximum strain relative to kMCL samples. As a result, PL samples plastically deform at a lower strain relative to kMCL and will fail at a lower strain. To summarize, both graft types withstand similar maximum stress, but since PL tendon is a stiffer material relative to kMCL, it withstands significantly lower strain before failure. The similarities in biomechanical properties between kMCL and PL suggest kMCL allografts are a suitable choice for further clinical investigator for UCLR.

Limitations of this study include a small sample size of 8 kMCL and 8 PL samples. In addition, the age of the PL donors was significantly greater than the MCL donors, which may affect some of the reported material properties. Furthermore, the average age of patients undergoing UCLR is 19.1 ± 4.1 years, demonstrating a significantly lower age of PL autografts used surgically [17]. The 50 mm mid-substance graft was selected to fit the customized jig to ensure consistency during mechanical testing and as a result we were unable to characterize the entire graft length, although Solon et al. previously demonstrated that the distal, mid, and proximal third of the PL tendon demonstrate similar mechanical properties [28]. The uniaxial tensile test performed in this study was subjected to a constant strain rate of 1% per second and may not characterize the tissues for all loading conditions, although strain rate dependence is relatively weak over physiological loading rates for ligaments and soft-tissues [5]. Lastly, the failing mechanism does not replicate the physiologic rotational moments placed on the elbow during throwing.
